# Reliability and validity of the Chinese version of the Sakata Eating Behavior Scale short form and preliminary analysis of the factors related to the score of the scale

**DOI:** 10.3389/fnut.2023.1076209

**Published:** 2023-03-08

**Authors:** Pu Ge, Xinpei Wang, Siying Gao, Jiaxin Liu, Yuyao Niu, Mengyao Yan, Siyuan Fan, Qiyu Li, Jinzi Zhang, Xiaonan Sun, Fei Wang, Yike Sun, Wenli Yu, Xinying Sun, Lian Yu, Yibo Wu

**Affiliations:** ^1^Institute of Chinese Medical Sciences, University of Macau, Macao SAR, China; ^2^Department of Medical Equipment, Peking University First Hospital, Beijing, China; ^3^Faculty of Education, Beijing Normal University, Beijing, China; ^4^Xiangya School of Nursing, Central South University, Changsha, China; ^5^Faculty of Arts and Humanities, University of Macau, Macao SAR, China; ^6^School of Health Policy and Management, Peking Union Medical College, Chinese Academy of Medical Sciences, Beijing, China; ^7^Department of Preventive Medicine, Yanjing Medical College, Capital Medical University, Beijing, China; ^8^School of Humanities and Health Management, Jinzhou Medical University, Jinzhou, China; ^9^Department of Social Science and Humanities, Harbin Medical University, Harbin, Heilongjiang, China; ^10^State Key Laboratory of Cognitive Neuroscience and Learning, Beijing Normal University, Beijing, China; ^11^College of Integrated Traditional Chinese and Western Medicine, Jining Medical University, Jining, China; ^12^School of Foreign Languages, Weifang University of Science and Technology, Weifang, China; ^13^School of Public Health, Peking University, Beijing, China; ^14^School of Public Health, Health Care System Reform and Development Institute, Xi'an Jiaotong University Health Science Center, Xi'an, China

**Keywords:** eating behavior, validity, reliability, cross-cultural adjustment, BFI-10, family health

## Abstract

**Background:**

The obesity rate in the Chinese population is increasing and there is a lack of short and reliable scales for measuring obesity-related eating behavior in China. The EBS-SF (Sakata Eating Behavior Scale short form) has only 7 entries and has shown good reliability in studies such as those in Japan.

**Objective:**

To translate the EBS-SF into Chinese, check its reliability, validity and explore the related factors.

**Method:**

The EBS-SF was translated into Chinese. 3,440 residents were investigated and 34 respondents were retested. Item analysis and reliability and validity tests were carried out. Personality characteristics, family health status and depression were investigated using the BFI-10, FHS-SF and PHQ-9 to investigate the factors associated with EBS-SF. The *t*-test, ANOVA and Pearson correlation was used to explore the related factors of its scores.

**Result:**

Among 3,440 residents, 1,748 (50.81%) were male and 1,692 (49.19%) were female; 1,373 (39.91%) were aged 36–50 years. All 7 items were qualified in the item analysis. As for reliability, the Cronbach's α was 0.870, the split-half reliability was 0.830, the test-retest correlation coefficient was 0.868. As for the structural validity, the standardized factor loadings were above 0.50, χ^2^ / df = 2.081,GFI = 0.999; NFI = 0.999; RFI = 0.996; RMSEA = 0.018, all qualified. The characteristics, personality, family health and depression were correlated with the score of the Chinese version of EBS short form.

**Conclusion:**

The structural validity and reliability of the Chinese version of the EBS-SF are good and it can be used as a measurement tool to evaluate the eating behavior of Chinese. The scores of the EBS-SF may be related to the sociological characteristics, personality, family health, and depression status.

## 1. Introduction

Over the past four decades, the dietary patterns of Chinese residents have undergone significant changes, with a rapid increase in the consumption of high-sugar and high-calorie foods and a rapid increase in the rates of overweight and obesity in the population. Obesity is increasingly becoming an important public health issue in China. As of 2019 statistics, the national prevalence of obesity is estimated at 6.8%, with obesity rates of 16.4% in adults (1). The development of obesity is closely related to uncontrolled eating behavior (2). Meanwhile, obesity is associated with the onset and progression of various chronic diseases, such as hypertension (3) and diabetes (4), which is very worrying. The prevalence of these chronic diseases rises with the obesity rate, thus significantly increasing the burden on the medical and public health systems of China.

The World Health Organization defines cut-off values for obesity based on the physical assessment such as body mass index (BMI): weight/height squared (kg/m^2^) (5, 6). Internationally, assessment methods for the obesity also involved such as nutrition assessment, exercise assesssment (4) and also the use of obesity genes. Obviously, it is not realistic to use any of these single indexes to describe the cause of obesity. And the above tools are more appropriate as therapeutic aids to identify obesity rather than exploring the causes of obesity. A valid assessment in terms of food intake as well as eating behavior seems to contribute to a better understanding of obesity and to give an active life management program.

In studies of the eating behavior of Chinese adults, it has been found that certain specific eating habits may constitute risk factors to obesity, such as the absence of a particular meal in a day. However, this may not be the only eating behavior that has an impact on obesity. There are complex interactions between eating behaviors and psychological, social and other factors. Related research confirms that body fat levels are closely related to eating behavior and that obesity may be driven by diet-related behavioral factors as well as pre-existing environmental and genetic factors (7). Therefore, a comprehensive assessment of eating behaviors is needed to identify specific potential risks for obesity.

Many studies exploring eating behaviors have used validated and reliable questionnaires that provide data tested in populations. For example, the Adult Eating Behavior Questionnaire (AEBQ) has been used all over the world. AEBQ is currently validated in Saudi, Poland, Portugal and China (8–11). The AEBQ has 35 items, using a five-point Likert scale involving eight subscales, which can be further divided into two dimensions of food approach and food avoidance behavior. Although this tool has been widely used and validated, the 35-item scale is very costly in terms of effort and time for the subjects and experimenters.

The thirty-item Sakata Eating Behavior Scale widely used in Japan is divided into seven dimensions: concern cognition of constitution, motivation for eating, substitute eating and drinking, feeling of satiety, eating style, meal contents, and eating rhythm abnormalities (12). Higher scores indicate poorer eating behaviors, which exacerbate obesity. The scale is used in some hospitals in Japan to assess eating behaviors to help patients change their eating behaviors and carry out obesity treatment.

The EBS short form was simplified from the 30-item EBS based on item response theory (13). Among the instruments measuring eating behavior associated with obesity, this is a much shorter scale, containing only seven items from the seven original dimensions. This means that the scale can be used in practice with less time and effort on the part of the user. The total score of the short scale is strongly correlated with the original scale (*r* = 0.93, *P* = 0.001). The EBS short form was validated in 1,032 Japanese adults aged 20–59 years and confirmed its validity.

In China, there is a lack of short scales that can be used in large cross-sectional surveys and identify more potential obesity problems. The EBS short form could be a more useful tool to measure Chinese eating behaviors and help Chinese obese patients to control their eating behavior. Considering the interoperability of Asian food cultures, the use of a simplified Chinese version of the EBS short form in China would be more culturally advantageous than the original scale developed and validated in countries outside of Asia. The objective of this study was to translate the simplified Chinese version of the EBS short form, check its reliability and validity in China and explore the possible influences on its scores.

## 2. Methods

This study is derived from a large cross-sectional study that callled “2021 China Family Health Index Investigation” (14). The data used in this study are a subset of this national study. The survey is based on multi-stage sampling across the country. When selecting cities, all the provincial capital cities of provinces and autonomous regions, as well as municipalities in China were firstly included. Later, the random-number table was applied to randomly select the non-provincial capital cities of all provinces and autonomous regions in the country. Finally, 120 cities were selected within China. During the second phase of sampling, the population of each city was stratified according to gender, age, and urban-rural distribution, and the sample size of each stratum was 100 people, which was determined according to the demographic characteristics of the “Seventh National Census in 2021”. Convenience sampling was carried out on the premise of meeting quota requirements. After the completion of the sampling, with the favor of the investigator recruited in each city, the investigation was conducted from 10th July 2021 to 15th September 2021. In detail, investigators of each city used the online questionnaire star platform (https://www.wjx.cn/) to distribute questionnaires one-on-one and face-to-face with people in their cities. Then, after the investigator entered the questionnaire number, respondents would complete the questionnaire by clicking on the link. If the respondents held the ability to think but were not able to act to answer the questionnaire, the investigator would help finish the questionnaire based on the offered answers by the participants. After unified training, investigators recruited from various provinces and cities distributed questionnaires to respondents meeting the inclusion and exclusion criteria through field investigation. Prior to the investigation, the investigator would use consistent instructions to explain the research purpose to the respondents, emphasizing the anonymity of the research and obtaining the informed consent of the respondents. During the investigation, the respondents filled out the questionnaire by themselves and then handed it over to the investigator for inspection. If there were omissions or multiple elections, the investigator would communicate with the respondents on the spot whether a by-election or re-election is possible. After the questionnaires were collected, the questionnaires whose filling time was <2 min, incomplete filling and inconsistent filling content were excluded.

### 2.1. Instruments

#### 2.1.1. The EBS short form

The EBS short form was developed in 2017 by Tayama and Ogawa et al., using item response theory (IRT) based on the Sakata Eating Behavior Scale (EBS) (13). The scale consists of 7 items, including eating rhythm abnormalities, feeling of satiety, eating habits, cognition of constitution, meal content, substitute eating and drinking, and motivation for eating, and each item is scored on a 4-point scale (1 = strongly disagree, 2 = somewhat disagree, 3 = somewhat agree, 4 = strongly agree). The scores of the 7 items were summed up as the total score of the scale, and the higher the respondent's score on this scale, the worse the eating behavior of the respondent.

#### 2.1.2. Other scales

##### 2.1.2.1. The 10-Item short version of the Big Five Inventory (BFI-10)

The 10-Item short version of the Big Five Inventory (BFI-10) was applied to measure the personality characteristics of the respondents. The scale consists of 5 dimensions with 10 items, and each dimension contains 2 items, including Extraversion, Agreeableness, Conscientiousness, Neuroticism, and Openness, on a 5-point Likert-type scale ranging from 1 (totally disagree) to 5 (totally agree). The full score of each dimension is 10 points, and the higher the score of a personality trait of the respondents, the more significant the personality trait of the respondents is. Several studies have shown that the BFI-10 has good reliability and validity (15–17).

##### 2.1.2.2. The Short-Form of the Family Health Scale (FHS-SF)

The family health level of the respondents was measured by the Short-Form of the Family Health Scale (FHS-SF) (18). The scale was developed by Crandall and Weiss-Laxer et al., and the simplified Chinese version has been validated in the Chinese population by Wang et al. (19). The FHS-SF is derived from the Family Health Scale-Long Form (FHS-LF), and it includes 4 dimensions, which are family social and emotional health process, family healthy lifestyle, family health resources, and family external social support, with a total of 10 items. Each item is scored on a 5-point Likert-type scale, among which items 6, 9 and 10 were scored in reverse. The higher the respondents' score on this scale, the higher the family health of the respondents. In the study, the Cronbach's coefficient of the scale was 0.851.

##### 2.1.2.3. The Patient Health Questionnaire-9 (PHQ-9)

The Patient Health Questionnaire-9 (PHQ-9) was used to measure the depression level of the respondents (20). There are 9 items on the scale, and each item is scored on a 4-point Likert-type scale ranging from 0 (never) to 3 (nearly every day). A total score between 0 and 4 indicates no depression; a total score between 5 and 9 indicates possible mild depression; a total score between 10 and 14 indicates likely moderate depression; a score between 15 and 19 indicates that there may be moderate to severe depression; a total score between 20 and 27 indicates that there may be severe depression. The higher the respondents' score on this scale, the higher the level of depression the respondents may have. In the study, the Cronbach's coefficient of the scale was 0.940.

### 2.2. Research process

**Figure 1:**
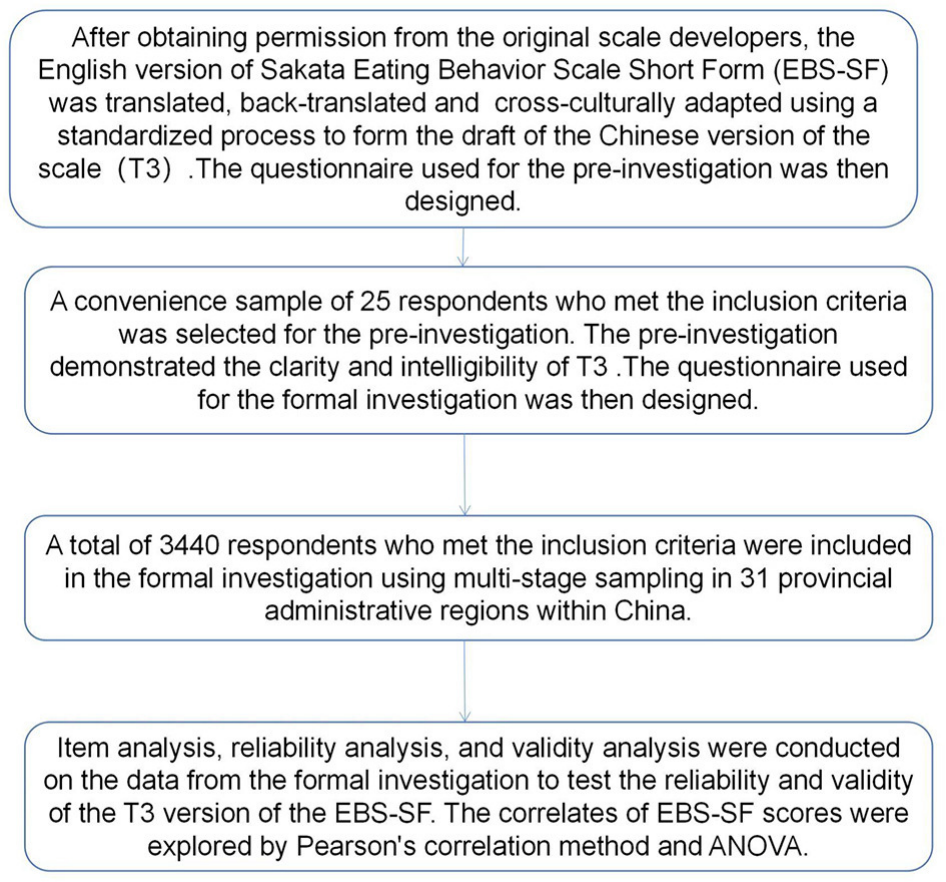
Research process of the study.

Figure 1 shows the entire process of this study.

#### 2.2.1. Scale translation

##### 2.2.1.1. Translation and back-translation of the scale

**Figure 2:**
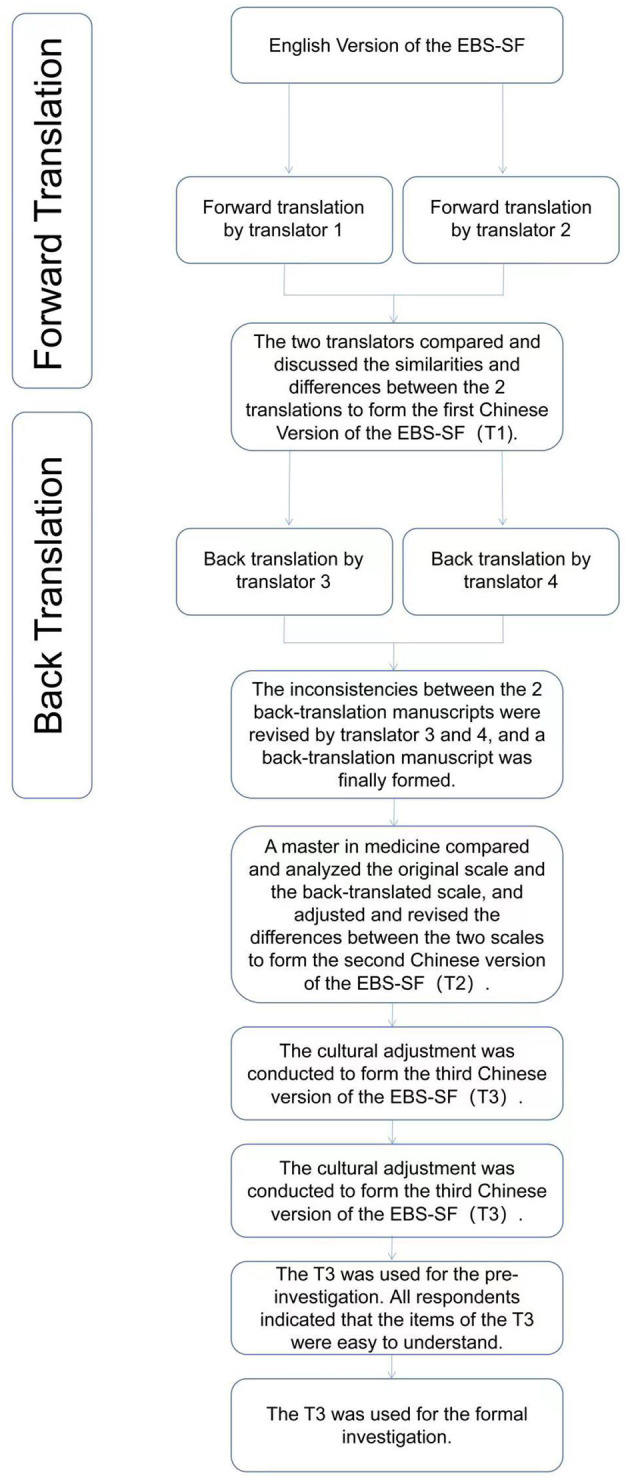
The process of the EBS-SF scale translation stage.

Figure 2 shows the process of the scale translation stage.

Translation stage. Expert consultation was used to qualitatively evaluate the content validity of the Chinese version of the scale (21).

Authorization for translation and use was obtained from the developers of the EBS short form, and the scale was translated independently by two masters (1 master in public health and 1 master in English-Chinese translation). Afterward, they compared and discussed the similarities and differences between the 2 translations to form the first draft (called: T1) of the Chinese version of the scale.

Back translation stage. The other 2 masters in English-Chinese translation were invited to back-translate the first draft T1 of the Chinese version of the scale separately without knowing the original English version of the EBS short form. The inconsistencies between the 2 back-translation manuscripts were revised, and a back-translation manuscript was finally formed.

Audit stage. A master in medicine compared and analyzed the original scale and the back-translated scale, and adjusted and revised the differences between the two scales to form the second draft (called: T2) of the Chinese version of the EBS short form.

##### 2.2.1.2. Cultural adjustment of the scale

The cultural adjustment group was composed of 16 experts (two in each field) from eight fields of psychology, sociology, social medicine, humanistic medicine, nursing, health education, health service management and behavioral epidemiology. All the members of the group are familiar with the process and methods of scale localization. According to the Guidelines for the process of cross-cultural adaptation of self-report measures (22), experts made judgments on the cultural adaptability of each item in the second draft T2 of the Chinese Version of the EBS short form, and made certain modifications based on Chinese expression habits without changing the original meaning. According to the suggestions of experts, the third draft (called: T3) of the Chinese Version of the EBS short form used in the pre-investigation stage was formed.

#### 2.2.2. Pre-investigation

The purpose of the pre-investigation was to finalize the wording of the Chinese version of the questionnaire. In May 2021, a convenient sampling of 25 respondents who met the inclusion criteria was used for a pre-investigation using the general data questionnaire and the third draft T3 of the Chinese version of the EBS short form. For the pre-investigation, we collected only general characteristics of the respondents and tested the respondents with the Chinese version of EBS-SF. Respondents were asked about the clarity and intelligibility of each item after completion and they all indicated that the items were easy to understand. Therefore, the third draft T3 of the Chinese Version of the EBS short form was used for the formal investigation.

#### 2.2.3. Formal investigation

##### 2.2.3.1. Participants of the formal investigation

###### 2.2.3.1.1. Inclusion/exclusion criteria

The questionnaire selected for this research was selected from the questionnaires in “2021 China Family Health Index Investigation” which met the requirements of this research.

The inclusion criteria of the participants were as follows: (1) Based on the original scale population the age ranged from 20 to 59 (13); (2) Had the nationality of the People's Republic of China; (3) China's permanent resident population with an annual abroad time ≤ 1 month; (4) Participate in the study voluntarily and fill in the informed consent form; (5) Participants can complete the questionnaire by themselves or with the help of investigators; (6) Participants can understand the meaning of each item in the questionnaire;

The exclusion criteria were as follows: (1) Persons with unconsciousness, eating disorders, or mental disorders; (2) Those who are participating in other similar research projects.

###### 2.2.3.1.2. Investigation method

The surveyors were recruited from online, and the surveyors conducted face-to-face interviews with the respondents and completed the online questionnaire on the spot for submission.

###### 2.2.3.1.3. The characteristics of the respondents

The characteristics of the participants that researchers collected comprised gender, age, marital status, educational level, Per capita monthly household income, current residence (urban / rural), region, occupational status, smoking status, drinking status.

#### 2.2.4. Quality control

The study conducted two rounds of pre-investigation before the formal investigation. Trained investigators distributed questionnaires to respondents and registered their codes one-on-one and face-to-face. Every Sunday evening during the investigation process, members of the research group communicated with the investigators to summarize, evaluate and give feedback on the questionnaires they collected. After the questionnaires were collected, two people conducted back-to-back logic checks and data screening. If singular values were found during data analysis, the original questionnaire must be found and checked with the investigator before proceeding to the next step of the analysis.

### 2.3. Statistical analysis

Data analysis was performed using SPSS 22.0 and AMOS 21.0. Statistical description of the sociological characteristics of the respondents was carried out using percentage, mean, etc. The correlation coefficient method, CITC method and extreme group method were used for item analysis The correlation coefficient method required those correlations of items with coefficients *r* < 0.35 or *P* > 0.50 associated with the total scale score be dropped; the extreme group method required that items with *t*-values obtained using independent sample *t*-tests in the high (highest 27%) and low (lowest 27%) subgroups be dropped if the differences were not significant (23). In addition, the CITC method requires that if the Cronbach's α of an item increases significantly after deletion, the item will be less internally relevant and should be deleted. Cronbach's α of internal consistency, split-half coefficient and test-retest reliability (intraclass correlation coefficient, ICC) were used for reliability analysis (24), and values ≥ 0.70 were considered to be good reliability.

In addition, confirmatory factor analysis was performed using AMOS 21.0 to test the structural validity of the scale. χ^2^ / DF < 3, GFI > 0.9, NFI > 0.9, RFI > 0.9 and RMSEA < 0.08 were used as the criteria for good structural validity of the model (25–27). The mean and standard deviation were used to describe the central tendency and dispersion degree of continuous variables. What's more, *t*-test or ANOVA was used for comparison between groups. After ANOVA, Bonferroni method was used for multiple comparisons. The pearson correlation method was used to analyze the correlation between the scores of other scales and the EBS short form. All data were tested with a two-sided test, and *P* < 0.05 was considered statistically significant unless otherwise stated.

## 3. Results

### 3.1. Characteristics of the participants and the score of the EBS short form

The 2021 “China Family Health Index Investigation” started from July 10, 2021 to September 9, 2021. A total of 11,688 questionnaires were distributed, 11,031 valid questionnaires were recovered, and a total of 3,440 cases were sampled based on the data from the 2021 “China Family Health Index Investigation”. Among 3,440 participants, 1,748 (50.81%) were male and 1,692 (49.19%) were female; 1,373 (39.91%) were people aged 36–50 years and the marital status of married people was the largest, with 2,278 (66.22%). There were 1,662 cases (48.31%) with a bachelor's degree or above and 1,667 cases (48.46%) with household per capita monthly income below 4,500 yuan. Nearly three-quarters of them live in urban areas, with 2,551 cases (74.16%). More than half of the cases were from the eastern part of the Chinese mainland, with 1,729 cases (50.26%) (Table 1).

**Table 1:** Sociological characteristics of the participants, the score of the EBS short form and Cronbach's coefficients for each subgroup.

**Item**		***N*** **(%)**	**Cronbach's αcoefficient**	**The EBS-SF scores**		
				**Mean** ±**SD**	***t*** **/** ***F***	***P***
**Gender**						
	Male	1,748 (50.81)	0.879	16.57 ± 4.618	0.749	0.454
	Female	1,692 (49.19)	0.862	16.46 ± 4.591		
**Age**						
	20–25	679 (19.74)	0.868	17.47 ± 4.649	30.227	< 0.001
	26–35	802 (23.31)	0.865	17.14 ± 4.524		
	36–50	1,373 (39.91)	0.866	16.17 ± 4.523		
	51–59	586 (17.03)	0.870	15.36 ± 4.514		
**Marital status**						
	Unmarried	1,052 (30.58)	0.869	17.48 ± 4.609	36.073	< 0.001
	Married	2,278 (66.22)	0.868	16.05 ± 4.534		
	Else (divorced or widowed)	110 (3.20)	0.845	16.98 ± 4.557		
**Educational level**						
	Junior school and below	649 (18.87)	0.866	16.46 ± 4.475	0.201	0.896
	Senior school and middle vocational school	596 (17.33)	0.859	16.42 ± 4.394		
	Junior college	533 (15.49)	0.884	16.54 ± 4.727		
	Bachelor and above	1,662 (48.31)	0.871	16.57 ± 4.690		
**Per capita monthly household income, yuan**						
	≤ 4,500 (663 dollars)	1,667 (48.46)	0.861	16.66 ± 4.402	2.197	0.111
	4,501–9,000 (663-1326 dollars)	1,216 (35.35)	0.873	16.30 ± 4.673		
	>9,000 (1,326 dollars)	557 (16.19)	0.885	16.55 ± 5.017		
**Place of residence**						
	Urban	2,551 (74.16)	0.868	16.51 ± 4.619	−0.176	0.860
	Rural	889 (25.84)	0.877	16.54 ± 4.565		
**Region**						
	Eastern	1,729 (50.26)	0.875	16.65 ± 4.702	6.173	0.002
	Central	979 (28.46)	0.866	16.68 ± 4.529		
	Western	732 (21.28)	0.861	15.99 ± 4.434		
**Occupational status**						
	Unoccupied	724 (21.05)	0.842	16.69 ± 4.249	16.895	< 0.001
	Employed	1,875 (54.51)	0.873	16.20 ± 4.614		
	Student	701 (20.38)	0.869	17.44 ± 4.648		
	Retired	140 (4.07)	0.910	15.19 ± 5.213		
**Smoking status**						
	Never smoking	2,651 (77.06)	0.872	16.39 ± 4.630	4.253	0.014
	Smoker	538 (15.64)	0.864	16.95 ± 4.529		
	Ex-smoker	251 (7.30)	0.864	16.90 ± 4.430		
**Drinking frequency**						
	Never drinking	1,846 (53.66)	0.882	16.12 ± 4.710	15.273	< 0.001
	Drinking, but not every week	974 (28.31)	0.842	16.88 ± 4.397		
	Drinking weekly	620 (18.02)	0.866	17.12 ± 4.496		

The Chinese and English versions of EBS-SF is shown in Supplementary Table 1. The *t*-test or ANOVA was used to test for differences in the EBS short form scores at each level of sociological variables. The results showed that there were significant differences in the scores of the EBS short form in participants of different ages, marital status, regions, occupational status, smoking status and drinking frequency (*P* < 0.05) (Table 1). The Bonferroni method was further used for the post hoc test of the results of ANOVA (see Supplementary Tables 2–7 for details). The scores of the older age group on this scale are significantly lower than that of the younger age group and the unmarried group were significantly higher than those of the married group; the scores of the western regions were significantly lower than those of the eastern and central regions and student's scores above other occupational status. As for smoking status and drinking frequency, never smoking scored lower than the smokers, and never drinking scored lower than drinking.

The mean score of the total score of the EBS short form was 16.52 ± 4.604 (Mean ± SD). See Table 2 for the score of each item in the EBS short form. The item with the highest mean score was “Item 3: eating fast” (2.52 ± 0.869), while the item with the lowest mean score was “item 7: when I buy food, I am satisfied when I buy more than I need” (2.21 ± 0.878). In each item, the choice with the largest number of people was “somewhat disagree” (items 2, 6, 7) or “somewhat agree” (items 1, 3, 4, 5), while the choice with the smallest number of people was “strongly agree”.

**Table 2:** Scores of each item in the EBS short form.

**Item**	**Scores (mea ±SD)**	**Median (lower quartile, upper quartile)**	**A *N* (%)**	**B *N* (%)**	**C *N* (%)**	**D *N* (%)**
1. Eat at all different times	2.31 ± 0.886	2 (2,3)	744 (21.63%)	1,121 (32.59%)	1,336 (38.84%)	239 (6.95%)
2. Do not feel satisfied unless I eat until full	2.25 ± 0.865	2 (2,3)	714 (20.76%)	139 (40.41%)	108 (31.66%)	247 (7.18%)
3. Eat fast	2.52 ± 0.869	3 (2,3)	497 (14.45%)	103 (30.17%)	153 (44.53%)	37 (10.84%)
4. Tend to gain weight more easily than others	2.51 ± 0.934	3 (2,3)	579 (16.83%)	102 (29.85%)	134 (39.04%)	49 (14.27%)
5. Like oily foods	2.36 ± 0.874	2 (2,3)	649 (18.87%)	1,191 (34.62%)	132 (38.63%)	271 (7.88%)
6.Eat if others around me are eating	2.36 ± 0.833	2 (2,3)	542 (15.76%)	135 (39.45%)	128 (37.44%)	253 (7.35%)
7.When buying food, I am not content unless I buy more than necessary	2.21 ± 0.878	2 (2,3)	805 (23.40%)	1,356 (39.42%)	1,040 (30.23%)	239 (6.95%)
Total score	16.52 ± 4.604	17 (14,20)				

A, strongly disagree; B, somewhat disagree; C, somewhat agree; D-strongly agree.

### 3.2. Item analysis and reliability and validity test of the EBS short form

#### 3.2.1. Item analysis

##### 3.2.1.1. Correlation coefficient method

The correlation analysis between each item and the total score of the questionnaire showed that each item score of the Chinese version of the EBS short form was significantly correlated with the total score of the scale, with correlation coefficients ranging from 0.694 to 0.794 (*P* < 0.001), above 0.35.

##### 3.2.1.2. CITC method

The Corrected item-total correlation (CITC) of the Chinese version of the EBS short form was all above 0.571, and the combination of the deleted Cronbach's α coefficient showed that the internal consistency coefficients did not change much after the deletion of the items (Table 3).

**Table 3:** Corrected item -total correlation of the simplified Chinese version of the EBS short form.

**Item**	**Cronbach's α after item deletion**
1. Eat at all different times	0.861
2. Do not feel satisfied unless I eat until full	0.844
3. Eat fast	0.858
4.Tend to gain weight more easily than others	0.861
5. Like oily foods	0.843
6. Eat if others around me are eating	0.846
7. When buying food, I am not content unless I buy more than necessary	0.845

##### 3.2.1.3. Extreme group method

The participants were ranked according to the total score of the scale, with 27% of the participants at both ends of the scale falling into the two extreme groups. The CR values of the high score group (≥ 19 points) and the low score group (≤ 14 points) were all above 3.0 (*P* < 0.001).

#### 3.2.2. Validity analysis

##### 3.2.2.1. Content validity

The content validity of the EBS short form was qualitatively evaluated by the expert consultation method. Experts made a qualitative evaluation of the relevance of each item of the Chinese version of the EBS short form to its measured content. 16 experts (two experts in each field of psychology, sociology, social medicine, humanistic medicine, nursing, health education, health service management, and behavioral epidemiology, all with master's or doctoral degrees). All experts agreed that each item in the scale could reflect the content to be measured, indicating that the Chinese version of the EBS short form had good content validity.

##### 3.2.2.2. Structural validity

The EBS short form consisted of only one dimension, so only validation factor analysis was used to test the structural validity of the scale, and the scale was validated according to the single factor structural model of the original scale, and the model was revised 8 times according to the Modified Index (MI). After the modification, the standardized factor loadings of the validation factor analysis were between 0.55 and 0.80, and the residuals were positive and significant.

**Figure 3:**
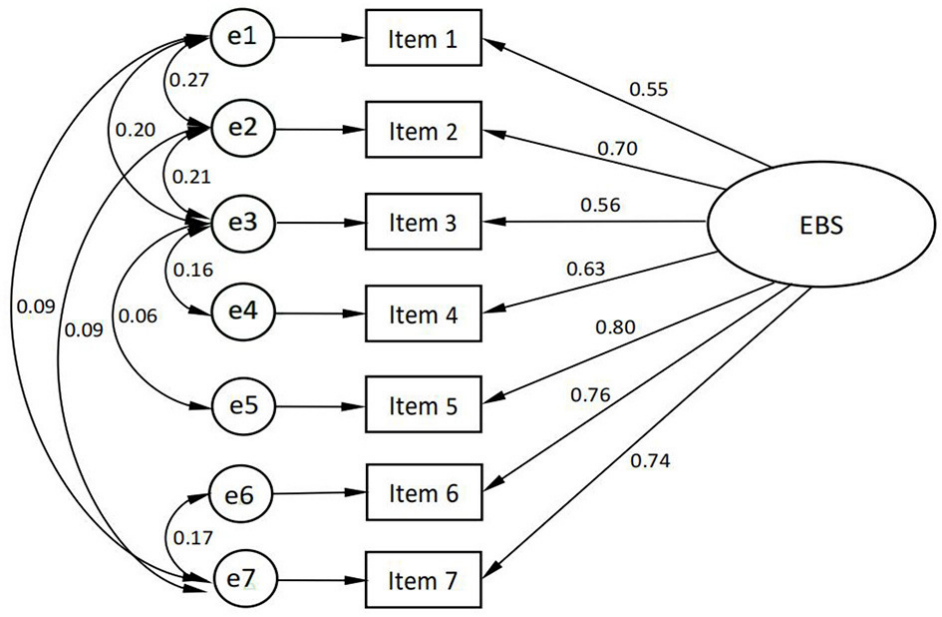
The validation factor analysis model for the Chinese version of the EBS short form.

The model fit indexes were χ^2^ / df = 2.081 < 3, GFI = 0.999 > 0.9, NFI = 0.999 > 0.9, RFI = 0.996 > 0.9, and RMSEA = 0.018 < 0.08, which is known from the fit indexes that the model structural validity is good and met the requirements. The results of the validation factor analysis are shown in Figure 3.

##### 3.2.2.3. Reliability analysis

The Cronbach's α coefficient of the Chinese version of the EBS short form was 0.870, and the split-half reliability was 0.830, which were all above 0.8. A total of 34 participants were selected by convenience sampling and retested at an interval of 2 weeks. We conducted a convenience sampling and retest reliability test based on respondents' willingness. The test-retest correlation coefficient (ICC) of the scale was 0.868, which was above 0.7.

### 3.3. The scores of the BFI-10, FHS-SF, and PHQ-9 and their correlation with the scores of the EBS short form

The specific scores of each dimension of BFI-10, FHS-SF and PHQ-9 scale of the participants are shown in Table 4. In BFI-10, the two dimensions with higher scores were agreeableness (7.02 ± 1.532) and conscientiousness (6.92 ± 1.621), and the two dimensions with lower scores were extraversion (6.27 ± 1.591) and neuroticism (5.77 ± 1.478). The overall family health status was good (37.99 ± 6.670). According to the assessment of the depression level of the participants according to their PHQ-9 scores, it was found that of the 3,440 participants, 1,572 had no depression (PHQ-9 score of 0 to 4), 1,171 had possible mild depression (PHQ-9 score of 5 to 9) There were 360 likely to have moderate depression (PHQ-9 score of 10–14), 250 likely to have moderate depression (PHQ-9 score of 15–19), and 87 likely to have severe depression (PHQ-9 score of 20–27).

**Table 4:** The scores of the BFI-10, FHS-SF, and PHQ-9 Scale of the participants and their correlation with the scores of the EBS short form.

**Scale**	**Number of entries**	**Score ranges**	**Mean ±SD**	**The scores of the EBS short form**
BFI-10				
Extraversion	2	2–10	6.27 ± 1.591	−0.015
Agreeableness	2	2–10	7.02 ± 1.532	−0.220^**^
Conscientiousness	2	2–10	6.92 ± 1.621	−0.264^**^
Neuroticism	2	2–10	5.77 ± 1.478	0.139^**^
Openness	2	2–10	6.42 ± 1.504	−0.031
FHS-SF	10	10–50	37.99 ± 6.670	−0.328^**^
PHQ-9	9	0–27	6.21 ± 5.700	0.396^**^

** Represents P < 0.01.

The scores of the EBS short form were significantly positively correlated with the scores of “neuroticism” (*r* = 0.139, *P* < 0.001) and the PHQ-9 (*r* = 0.396, *P* < 0.001). The scores of “agreeableness” (*r* = −0.220, *P* < 0.001), “conscientiousness” (*r* = −0.264, *P* < 0.001) and the FHS-SF(*r* = −0.328, *P* < 0.001) were significantly negatively correlated with the scores of the EBS short form, as shown in Table 4.

## 4. Discussion

### 4.1. The Chinese version of the simplified EBS short form has good reliability and validity

In the field of eating behavior research, this study obtained preliminary results in the context of providing a national sample of the Chinese population, and the psychometric characteristics and factor structure of the simplified Chinese version of the EBS short form. The equivalence between the Chinese version of the scale and the original scale was fully ensured through a rigorous scale introduction process, including translation, back translation, expert consultation, and prediction. The good results of item analysis and the good correlation among the items and the total score of the scale illustrate the good reliability of the Chinese version of the EBS short form, the good representation of the items of this scale and the ability to measure eating behavior effectively. After validation factor analysis, sufficient structural validity represented that the original single factor structural model agreed well with the Chinese version of the EBS short form data. The factor loadings of all items in each dimension of the English version of the EBS short form were above 0.6, and the standardized factor loadings of the Chinese version of the factor model were between 0.55 and 0.80, which are more consistent with the original scale. It is generally considered that the Cronbach's α of the total scale is above 0.80, and the test-retest reliability is above 0.7, which means that the reliability of the scale is good. Cronbach's α coefficient and test-retest reliability of the total scale met the measurement requirements, indicating that the Chinese version of the EBS short form has good internal consistency, high reliability, and temporal stability. EBS short form in English with Cronbach's α was 0.830, and Cronbach's α for this study was 0.870, which is close to that of the previous study (13).

### 4.2. Factors associated with the Chinese version of the EBS short form scores

The EBS short form looks at the differences in eating habits between obese and healthy individuals, with higher scores reflecting worse eating habits. Four aspects were analyzed: personal characteristics, interpersonal networks, personal behavior and social factors.

#### 4.2.1. Personal characteristics

##### 4.2.1.1. Personality traits

The Big Five Personality Inventory, a powerful model for measuring human personality traits, helps us to analyze differences in eating behavior in the population. Our study exploratively found that conscientiousness in the Big Five personality traits may have a significant negative impact on eating behavior, which is similar to the findings of Keller et al. (28). Conscientiousness can lead to more consumption of recommended foods and less consumption of non-recommended foods. In addition, agreeableness is negatively associated with poor eating behavior and relevant to, low emotional under-eating and low emotional overeating (29, 30). What's more, the present study showed a positive association between neuroticism and poor eating behavior, similar to previous studies (31). It could be that neuroticism is associated with emotional eating (32). Emotional instability, impulsiveness and poor self-control are not conducive to good eating habits.

##### 4.2.1.2. Age

In this study, age was viewed as a categorical variable, the significantly higher scores on eating behavior among those under 35 years of age in this study compared to those over 35 years of age may be related to the fact that emotional eating is more prevalent in younger age groups (33). Younger populations have a stronger tendency to be more impulsive to attractive food stimuli, have lower self-regulation and seek higher pleasure, thus increasing the likelihood of undesirable eating behavior (30, 34).

#### 4.2.2. Interpersonal networks

##### 4.2.2.1. Marital status

The results showed that married residents scored lower on the EBS short form than unmarried, which is consistent with a previous study (35). This may be because people are encouraged and supervised by their spouses after entering marriage, which promotes healthy eating behavior (36).

##### 4.2.2.2. Family Health

This study showed a significant negative correlation between the EBS short form scores and FHS-SF scores, which is consistent with previous studies (35). A good family health function not only provides sufficient family health resources to help families better meet their daily needs and perform their functions but also promotes emotional communication between family members, therefore, it is helpful to develop good eating habits (18, 37).

#### 4.2.3. Personal behavior

##### 4.2.3.1. Lifestyle

Lifestyles such as smoking and drinking were associated with high EBS short-form scores. This may be related to the fact that alcohol consumption stimulates appetite and even leads to binge eating (38, 39). Quitting smoking may lead to uncontrolled eating as a result of quitting and thus enhancing the stimulatory response to food. Nicotine, on the other hand, has a suppressive effect on one's appetite, which could explain the relationship between smoking and disordered eating habits, for example, adolescents may smoke in the hope of losing weight (40).

##### 4.2.3.2. Emotional processing

We also explored whether depression was associated with eating behavior, with PHQ-9 scale scores showing a significant positive correlation with eating behavior scores. It may be because depression affects a person's motivation to make food choices, thus reducing the likelihood of choosing healthy meals (41). Although this result is consistent with the findings of a larger number of studies on eating behavior, more research is needed to confirm whether depression is associated with eating behavior in the broad sense (42–44).

#### 4.2.4. Socio-demographic characteristics

##### 4.2.4.1. Occupational status

Poor eating behavior was more pronounced in the student group in this study compared to other occupational states. This is similar to the conclusion of Stok (45) that eating behavior usually becomes unhealthy during the transition from adolescence to young adulthood. When students start college, they are faced with new pressures and a lack of time for activity and financial ability, which can have a strong impact on their eating habits and willingness to engage in healthy behaviors (46).

##### 4.2.4.2. Region of residence

Those in the central and eastern regions of the country performed less well in terms of eating behavior than those in the western regions, and some studies have found that residents living in developed regions are more likely to have eating behavior disorders, which is consistent with the results of this study (47). The pace of life in economically developed areas is fast, and there are more diets available for people to choose. In addition, food-related takeaway and express delivery services are more convenient, and poorer eating behavior may be related to these factors.

### 4.3. Limitations

In this study, we did not set a scalar scale, so we could not give the scalar validity of this scale and other validated scales, which is one of the limitations of this study. The simplified scale has only seven items, and the answer format with fewer items allows us to complete the test on a larger population. Due to practical difficulties in secondary data collection, the retest reliability of this study was based on a convenience sampling method without using the original study sample, which may be a source of bias.

## 5. Conclusion

In conclusion, this study demonstrates that the simplified Chinese version of the EBS short form has good psychometric properties and is a valid and reliable tool for assessing eating behavior in Chinese adults. This tool is easy to use in population-based studies because of its self-reported nature and brevity. This study also explored the relationship between personal characteristics such as personality traits or depression status and eating behavior. Although there are some limitations, this study preliminary validated the reliability and validity of the simplified Chinese version of the EBS short form in a national Chinese sample. Future research should focus on the mechanism by which various related factors affect eating behavior, and should also focus on the relationship between the EBS short form score and obesity-related indicators such as BMI, also with the differences in eating behavior between obese and normal people.

## Data availability statement

The raw data supporting the conclusions of this article will be made available by the authors, without undue reservation.

## Ethics statement

The studies involving human participants were reviewed and approved by Institutional Review Committee of Jinan University, Guangzhou, China (JNUKY-2021-018). The patients/participants provided their written informed consent to participate in this study. Written informed consent was obtained from the individual(s) for the publication of any potentially identifiable images or data included in this article.

## Author contributions

LY and YW: directed and supervised the project. PG, XW, LY, and YW: designed research and had primary responsibility for the final content. PG, XW, and JL: drafted the first manuscript. PG, SG, XiaS, FW, and YW: scale translation. PG, SG, WY, YS, and YW: collected the data. PG, WY, YS, and YW: performed the statistical analysis. PG and XW: interpreted the results and wrote the manuscript. PG, XW, XiaS, FW, YN, MY, JZ, SF, QL, XinS, LY, and YW: provided critical revision for important intellectual content of the manuscript. All authors read and approved the final manuscript.
