# Supplementary material for: Reliability and validity of the Chinese version of the Sakata Eating Behavior Scale short form and preliminary analysis of the factors related to the score of the scale
Source: Front Nutr. 2023 Mar 8;10:1076209. doi: 10.3389/fnut.2023.1076209 (PMC10031001; doi:10.3389/fnut.2023.1076209)
Supplement: Supplementary file 1 [file Data_Sheet_1.docx]

| **Supplementary Table 1 English and Chinese versions of EBS-SF** | |
| --- | --- |
| Language of the scale | Full text of the scale |
| English | For each item, please answer from 1 (strongly disagree) to 4 (strongly agree).  □strongly agree, □ somewhat agree, □ somewhat disagree, □strongly disagree   1. Eat at all different times. 2. Do not feel satisfied unless I eat until full. 3. Eat fast. 4. Tend to gain weight more easily than others. 5. Like oily foods. 6. Eat if others around me are eating. 7. When buying food, I am not content unless I buy more than necessary. |
| Chinese | 请选择适当的一个选项来评价您对以下陈述的认同程度：  □非常不同意 □ 有些不同意 □ 有些同意 □非常同意   1. 没有固定的用餐时间 2. 只有吃十分饱才满足 3. 吃饭速度快 4. 我认为我比别人更容易长胖 5. 喜欢高油脂的食物 6. 如果我周围的人在吃东西的话，我也吃 7. 我购买食物的时候，购买的数量超过我的需要，我才满意 |

**Supplementary Table 2 Post hoc test of the EBS short form in different age groups**

| Total score  Group | A-B | A-C | A-D | B-C | B-D | C-D |
| --- | --- | --- | --- | --- | --- | --- |
| MD | 0.322 | 1.298 | 2.103 | 0.976 | 1.781 | -0.805 |
| *P* | 1.000 | <0.001 | <0.001 | <0.001 | <0.001 | 0.002 |

Note: A represents 20-25 years old, B represents 26-35 years old, C represents 36-50 years old, and D represents 51-59 years old.

**Supplementary Table 3 Post hoc test of** **the EBS short form in different marital status**

| Total score  Group | A-B | A-C | B-C |
| --- | --- | --- | --- |
| MD | 1.431 | 0.497 | -0.934 |
| *P* | <0.001 | 0.829 | 0.108 |

Note: A represents unmarried, B represents married, C represents other (divorced, widowed).

**Supplementary Table 4 Post hoc test of the EBS short form in different regions**

| Total score  Group | A-B | A-C | B-C |
| --- | --- | --- | --- |
| MD | -0.033 | 0.660 | 0.693 |
| *P* | 1.000 | 0.003 | 0.006 |

Notes: A represents eastern, B represents central, and C represents western.

**Supplementary Table 5 Post hoc test of the EBS short form in different occupational status**

| Total score  Group | A-B | A-C | A-D | B-C | B-D | C-D |
| --- | --- | --- | --- | --- | --- | --- |
| MD | 0.494 | -0.749 | 1.508 | -1.243 | 1.014 | 2.257 |
| P | 0.082 | 0.012 | 0.002 | <0.001 | 0.069 | <0.001 |

Note: A represents unoccupied people, B represents employed, C represents students, and D represents retired people.

**Supplementary Table 6 Post hoc test of the EBS short form in different smoking status**

| Total score  Group | A-B | A-C | B-C |
| --- | --- | --- | --- |
| MD | -0.561 | -0.506 | 0.055 |
| P | 0.030 | 0.288 | 1.000 |

Note: A represents never smoking, B represents smokers, and C represents ex-smokers.

**Supplementary Table 7 Post hoc test of the EBS short form in different drinking frequency**

| Total score  Group | A-B | A-C | B-C |
| --- | --- | --- | --- |
| MD | -0.758 | -0.999 | -0.241 |
| *P* | <0.001 | <0.001 | 0.922 |

Note: A represents never drinking, B represents drinking but not every week, and C represents drinking every week.
